# Approach to investigation and management of proteinuria in pregnancy

**DOI:** 10.1016/j.clinme.2024.100281

**Published:** 2024-12-26

**Authors:** Isabela Bertoni, Sion Williams

**Affiliations:** a Women's Centre, John Radcliffe Hospital, Oxford University Hospitals, Headley Way, Headington, OX3 9DU; b Royal Berkshire Hospital, Reading, RG1 5AN

**Keywords:** Pregnancy, Proteinuria, Nephrology, Hypertension, Obstetric medicine

## Abstract

Pregnancy leads to significant changes in renal physiology, which result in increases in glomerular filtration rate (GFR) and enhanced protein excretion. These changes may continue in the postnatal period and might be observed for 5–6 months after birth. Once confirmed, proteinuria warrants investigation and close surveillance. Clinicians should establish the level of excretory kidney function and the presence or absence of proteinuria at booking/diagnosis. A history of proteinuria, PET and anti-hypertensive requirements in previous pregnancies is a helpful guide to what to expect in the current pregnancy. Maternal physiological adaptations mean that yet-undiagnosed kidney disease is unmasked during pregnancy. New onset of proteinuria before 20 weeks’ gestation (with or without kidney impairment) suggests known or previously undetected kidney disease. As pregnancy evolves, hyperfiltration may lead to increasing proteinuria, posing a diagnostic challenge in the diagnosis and recognition of pre-eclampsia. This article was written as a guide for the evaluation and management of proteinuria in pregnancy, as well as appreciating diagnostic dilemmas.

**Key points**

1. Women with chronic hypertension, CKD, previous history of gestational hypertension, gestational proteinuria and pre-eclampsia should undergo pre-conception counselling.
2. Proteinuria detected before 20 weeks’ gestation represents pre-existing kidney disease.
3. Consider the diagnoses of PET after 20 weeks’ gestation in those with *de novo* proteinuria / worsening pre-existing proteinuria and uncontrolled hypertension.
4. Offer thromboprophylaxis for those with nephrotic range proteinuria.
5. Pregnancy can accelerate kidney disease, accelerating the need for dialysis and/or transplantation – particularly those with CKD stages 3a–5.

## Protein excretion and normal pregnancy

Pregnancy leads to significant changes in renal physiology. Systemic vasodilatation and volume expansion result in a 50% increase in glomerular filtration rate (GFR) and changes in tubular function and protein handling, leading to enhanced protein excretion. These changes may continue in the postnatal period and might be observed for 5–6 months after birth.^1^

In healthy, non-pregnant people, urine excretion does not exceed 150 mg in 24 h. In a healthy pregnancy, renal and maternal adaptation can increase protein excretion from pre-pregnancy levels, but this should not exceed 300 mg in 24 h.^2^

## Definition and detection of proteinuria

Urinalysis to screen for proteinuria is frequently performed during antenatal checks.

Proteinuria is the presence of more than 300 mg protein in the urine over 24 h (spot urine protein:creatinine ratio (uPCR) of more than 30 mg/mmol), applicable to both pregnant and non-pregnant states. 24-hour urine collection for protein quantification is not required. A prospective study of 270 healthy pregnant women showed that the upper ‘normal’ limit of protein excretion was 260 mg/day (uPCR 26 mg/mmol).^3^

## Assessment of kidney function in pregnancy

When pregnant, serum creatinine should be used instead of a quoted estimated GFR (eGFR).^4^

In healthy pregnant women, creatinine falls in the first trimester, reaches a nadir and plateau in the second trimester, gradually rises in the third trimester and, on average, returns to pre-pregnancy baseline by 18 weeks post-partum. Harel *et al* described this in a graph, which readers are encouraged to see.^4^

## Nephrotic range proteinuria in pregnancy

Nephrotic range proteinuria is defined as a urine albumin:creatinine ratio (uACR) of more than 2.5 g/24 h (>250 mg/mmol) or a uPCR of >3 g/24 h (>300 mg/mmol).^5^ Serum albumin might be lower due to physiological haemodilution, but the extent of this is incredibly variable.

The general principles of managing nephrosis are as follows.

• Salt restriction should be avoided in pregnancy.
• Serum albumin is not a reliable indicator of nephrosis.
• Thromboprophylaxis with prophylactic-dose low-molecular-weight heparin (LMWH) should be offered antenatally and continued for at least 6 weeks postnatally. Women who commence anticoagulant therapy in pregnancy should have a birth plan.
• Diuretics are not routinely recommended due to the risk of oligohydramnios, but should be considered on a case-by-case basis in conjunction with the obstetric physician or nephrologist.
• Some women with primary kidney disease may either start, or already be taking, immunosuppressive agents such as:
  ○ calcineurin inhibitors (eg tacrolimus, ciclosporin)
  ○ azathioprine
  ○ rituximab is now being used more frequently in early pregnancy. It should be avoided in the second and third trimesters to avoid neonatal B-cell depletion
  ○ prednisolone.
• Women on steroids or calcineurin inhibitors have a greater risk of gestational diabetes and should be offered an oral glucose tolerance test (OGTT).

Pre-eclamptic women with singleton pregnancies and new-onset proteinuria levels 300–499 mg/24 h are at higher risk of severe hypertension, earlier delivery and fetal growth restriction than those with gestational and chronic hypertension. Those with proteinuria above 500 mg/24 h have an even greater risk of adverse pregnancy outcomes (pre-term delivery, caesarean section, or requiring magnesium sulphate).^6^

## Causes of proteinuria in pregnancy

### Pre-eclampsia (PET)

PET is defined as new onset of hypertension (BP ≥140 mmHg systolic or ≥ 90 mmHg diastolic on more than two occasions) after 20 weeks, with one or more of the following^7^:

• new onset proteinuria
• maternal organ dysfunction:
  ○ kidney impairment (serum creatinine >90 micromol/L)
  ○ liver involvement (ALT or AST more than twice the upper limit of normal) with or without right upper quadrant or epigastric pain
  ○ neurological complications (eclampsia, altered mental status, visual disturbance, stroke, myoclonus, papilloedema, severe headaches)
  ○ haematological complications:
    ■ platelets <150 × 10^3^/L
    ■ disseminated intravascular coagulation (DIC)
    ■ haemolysis
• uteroplacental dysfunction:
  ○ fetal growth restriction (<10th centile)
  ○ abnormal umbilical artery dopplers
  ○ placental abruption
  ○ stillbirth.

Around one in 10 women with PET do not become proteinuric – the presence of proteinuria is not mandatory for its diagnosis. Conversely, it is the commonest cause of nephrotic-range proteinuria in pregnancy.^8^

Women with one or more high-risk factors or more than one moderate-risk factor are advised to take 75–150 mg aspirin once daily from 12 weeks until 36 weeks’ gestation. They may be offered increased fetal surveillance at 28, 32 and 36 weeks (Table 1).^9^

**Table 1:** Obstetric and Medical risk factors for PET.

Obstetric risk factors	Medical risk factors
Maternal age (>40 years)^	Pre-existing hypertension*
Nulliparity^	Chronic kidney disease (CKD)*
Multiple pregnancy^	Diabetes mellitus (DM)*
Pregnancy interval >10 years^	Connective tissue disorders*
Hypertensive disease in previous pregnancy*	Anti-phospholipid syndrome*
Family history of pre-eclampsia (first-degree relative)^	BMI > 35 kg/m^2^ at booking^
IVF (particularly with donor eggs)	Sickle cell disease

* High risk factor and ^ moderate risk factor according to the National Institute for Health and Care Excellence (NICE).^9^

PET is characterised by an imbalance between pro-angiogenic factors (placental growth factor (PlGF)) and anti-angiogenic factors (soluble fms-like tyrosine kinase-1 (sFlt-1)). PlGF levels are variable according to gestation; however, low levels are indicative of placental dysfunction (Table 2).^10^ In women with singleton pregnancies with suspected PET between 24 to 36+6 weeks, the serum sFlt-1/PlGF ratio has a high negative predictive value and can be a valuable adjunct to rule out PET. It can help risk stratify susceptible women of developing PET within 7–14 days after the test, prompt enhanced surveillance of those considered higher risk, and prevent unnecessary admissions for lower-risk women (Table 3).^11^

**Table 2:** Recommended cut-offs for PlGF testing PlGF levels, National Institute for Health and Care Excellence (NICE). Levels vary by gestational age, with the lower limit of normal ranging from 141 pg/mL at 30 weeks**’** gestation to 23 pg/mL at term.^10^

PlGF level (pg/mL)	Interpretation
<12	Highly abnormal – suggestive of severe placental dysfunction and increased risk of pre-term delivery
12–100	Abnormal – suggestive of placental dysfunction and increased risk of pre-term delivery
≥100	Normal – suggestive of no placental dysfunction and unlikely to progress to delivery within 14 days

**Table 3:** Correlation between sFlt/PlGF ratio and the risk of developing PET within 7 and 14 days.^11^

sFlt/PlGF ratio	Risk of PET in the next 7 days	Risk of PET in the next 14 days
≤38	0.4%	1.2%
>38 and ≤85	20%	26.7%
>85	54.7%	67.9%

Depending on gestation and degree of end-organ damage, the benefits and risks of continuing versus ending the pregnancy by expediting birth should be discussed – particularly for preterm PET.^9^ A plan must include:

• the timing and nature of fetal monitoring while the pregnancy continues
• fetal and maternal indications for expediting birth and a plan for antenatal corticosteroids (to support fetal lung maturation)
• discussion regarding place of birth, considering local anaesthetic and neonatology support and expertise
• follow-up in the postpartum period for cardiometabolic assessment and optimisation in either primary or secondary care.

### Pre-existing hypertension

This is defined as BP ≥140/90 mmHg on two separate occasions – diagnosed before pregnancy or before 20 weeks’ gestation. Around a quarter of women with pre-existing or chronic hypertension will develop pre-eclampsia, around 28% will require a preterm birth, 17% will have a birthweight <2,500 g and the risk of placental abruption and perinatal death is also greater.^12^

Once established on medications, target BP of ≤ 135/85 mmHg. ACE inhibitors and angiotensin II receptor blockers (ARB) have well-established antiproteinuric effects. Due to an increased risk of congenital abnormalities, they should be stopped either before conception or with the first positive pregnancy test. Alternative agents that are safe in pregnancy include labetalol, nifedipine, amlodipine, methyldopa, doxazosin and hydralazine.

As pregnancy progresses, pre-existing proteinuria may increase with the physiological increase in GFR.

The possibility of PET after 20 weeks must be considered if there is doubling of antihypertensive agents and/or rise in proteinuria (a doubling of uPCR or uACR compared to early pregnancy). PlGF-based testing between 24 and 36+6 weeks may be useful in this setting. PlGF is renally excreted and may be artificially low with higher creatinine levels, and must be interpreted with caution.^13^

Women should be advised to have a minimum yearly review of their blood pressure and kidney function, a check of proteinuria (which can be quantified and compared), and an ECG – due to this cohort’s heightened risk of cardiovascular disease.

### Gestational proteinuria

Gestational proteinuria is defined as isolated proteinuria arising at or after 20 weeks’ gestation in a woman without PET or primary kidney disease, with an incidence of 0.55–7.0% of pregnancies.^14^ It often resolves by 3 months after birth and is, therefore, a retrospective diagnosis.

Retrospective analyses have shown that, of those women with PET, isolated new-onset proteinuria without hypertension was the initial presentation in 13–25% of women^15^ – highlighting that this group of women require enhanced surveillance antenatally.

After birth, women should have a repeat uPCR and BP assessment at 12 weeks. If there is ongoing proteinuria beyond 12 weeks, please discuss with nephrology.

### Chronic kidney disease

Chronic kidney disease (CKD) is classified according to the level of excretory kidney function and the degree of proteinuria (see Table 4).^15^ Common causes of proteinuric CKD in pregnant women include hypertension, diabetic nephropathy, SLE and IgA nephropathy.

**Table 4:** Kidney Disease - Improving Global Guideline (KDIGO) for the Evaluation and Management of Chronic Kidney Disease. Prognosis of CKD by GFR and albuminuria categories. Green: low risk (if no other markers of kidney disease, no CKD); Yellow: moderately increased risk; Orange: high risk; Red: very high risk. GFR, glomerular filtration rate.^16^

The assessment and management of women with CKD and childbearing potential should start before conception.

Women with CKD have an increased risk of PET, fetal growth restriction, preterm birth, the neonate requiring admission to intensive care, and birth by caesarean section.^17^

Pregnancy may accelerate the progression of kidney impairment – particularly so in women who conceive but are in the latter stages of CKD (3a–5). Women with uncontrolled SLE with even mild kidney impairment can have a much more precipitous decline in kidney function.^18^

The presence of proteinuria increases the risk of having a baby with a low birth weight, whereas the presence of hypertension increases the chance of pre-term delivery.^17^

In pregnancy, the following factors are associated with deteriorating CKD:^17^

• difficult to control hypertension
• heavy proteinuria
• a < 10 % fall in serum creatinine in the first trimester.

#### Antenatal management of proteinuria

##### De novo proteinuria before 20 weeks***’*** gestation in those without a renal diagnosis

This represents new or previously undetected kidney disease (Table 5). For assessment, refer to Table 4.

**Table 5:** Key factors in the history and diagnostic work-up of proteinuria in pregnancy.

**Kidney function and proteinuria quantification**	Current and historical trend of creatinine Current and previous episodes of hypoalbuminaemia and proteinuria
**New nephrotic range, send**	Antibodies to the phospholipase A2 receptor (PLA2R antibodies) Viral serology should be available from booking HbA_1C_
**If persistent microscopic haematuria (≥3+ blood on dipstick) send a renal acute screen**	ANCA ANA Complement Immunoglobulins Rheumatoid factor (RhF) Anti-glomerular basement membrane antibody(anti-GBM) and myeloma screen are rarely needed. Discuss with local nephrology team
**Assess fluid status**	BP Weight JVP Peripheral oedema? Pulmonary oedema?
**Previous pregnancies**	Gestational hypertension and medication requirement Pre-eclampsia Gestation and mode of birth (vaginal, instrumental, C-section) and reasons why
**Screen for diabetes**	Osmotic symptoms (polyuria, polydipsia) Other microvascular disease (retinopathy, neuropathy) Consider early OGTT if there are risk factors HbA_1c_ >48 mmol/mol in the first trimester is suggestive of pre-existing diabetes mellitus
**Screen for SLE and connective tissue disorders (CTDs)**	Alopecia Mouth ulcers Rashes Arthralgia Previous clots/miscarriages Chest pain
**Family history**	Kidney disease Hypertension Diabetes Pre-eclampsia
**Medication history**	
**Renal ultrasound**	To assess morphology and look for signs of chronicity Useful as a benchmark, in case renal biopsy is contemplated in the future

##### Increasing proteinuria before 20 weeks in women with known renal disease

If there is rapidly progressive kidney dysfunction alongside increasing proteinuria, seek urgent advice from obstetric medicine / nephrology teams, as there may be fetal indications to commence haemodialysis earlier if the urea reaches 17–20 micromol/L.

In women with lupus, screen for signs and symptoms of a flare. A rise in creatinine and/or proteinuria associated with pancytopenia, rising dsDNA, low complement and a high CRP may be indicative of a flare. This should be discussed with the nephrology team.

Those with diabetic nephropathy are at a heightened risk of deteriorating kidney function and increasing proteinuria in the second and third trimesters – and distinguishing between disease progression and superimposed PET may be challenging.

##### Proteinuria after 20 weeks***’*** gestation

Consider the diagnosis of superimposed PET in CKD:^5^

• in a woman with non-proteinuric CKD, if she develops new hypertension and proteinuria or maternal organ dysfunction after 20 weeks’ gestation
• in a woman with proteinuric CKD, if she develops new hypertension or maternal organ dysfunction after 20 weeks’ gestation
• in a woman with chronic hypertension and proteinuria, if she develops maternal organ dysfunction after 20 weeks’ gestation.

#### Indications for a kidney biopsy in pregnancy

A kidney biopsy can be considered in the first or early second trimester, and only if a tissue diagnosis will change management. The main indications for proceeding with a biopsy include:^1^

• new nephrotic syndrome, rapidly deteriorating kidney function, or a haematoproteinuric acute kidney injury – with an unclear diagnosis or uncertainty about how best to manage
• to optimise disease management where a change in immunosuppressive therapy or plasma exchange may help prolong the pregnancy up to 32 weeks
• suspected acute graft rejection in a patient with a kidney transplant.

## Declaration of competing interest

The authors declare that they have no known competing financial interests or personal relationships that could have appeared to influence the work reported in this paper.
